# Unilateral baseball pitching: morphological and functional adaptations in the neck muscles

**DOI:** 10.3389/fspor.2025.1452412

**Published:** 2025-02-10

**Authors:** Leila Rahnama, Ceren Acik, Christine Dy, Stefan Keslacy

**Affiliations:** School of Kinesiology, California State University, Los Angeles, Los Angeles, CA, United States

**Keywords:** neck muscles, baseball, muscle adaptation, functional asymmetry, strength, proprioception

## Abstract

**Background:**

Functional asymmetry and muscle imbalances are recognized as contributors to injury risk in athletes. Sports with repetitive unilateral movements such as baseball pitching can lead to adaptations in shoulder and scapular muscles. There is a lack of research on whether these movements result in neck muscle alterations. Understanding potential asymmetries in neck musculature could provide valuable insights into athletes’ performance and injury prevention strategies.

**Methods:**

A total of 14 collegiate baseball pitchers and 15 controls voluntarily participated in this study. Bilateral dorsal neck muscle thickness, stiffness, neck range of motion (ROM), neck repositioning error, and extensor strength were measured, and the asymmetry between the two groups was compared. Rehabilitative ultrasound imaging was used to assess muscle thickness and stiffness. An inclinometer and a dynamometer were utilized to evaluate neck ROM and strength, respectively.

**Results:**

The mean age of the baseball pitchers and controls was 21.86 ± 1.6 and 25.87 ± 5.10 years, respectively. A significantly greater thickness of the splenius capitis on the non-dominant side was observed in baseball pitchers [*p* = 0.029, effect size (ES) = 0.857], whereas controls demonstrated symmetrical muscle thickness in all dorsal neck muscles. Pitchers exhibited higher neck extensor maximal voluntary contraction compared to controls (*p* = 0.017, ES = 0.926). Controls showed more bilateral differences in muscle stiffness in the splenius capitis and the semispinalis cervicis, although statistical asymmetry was not demonstrated.

**Conclusion:**

The cervical multifidus muscles showed bilateral symmetry despite the unilateral throwing motion in baseball pitching. However, unilateral neck rotation toward the non-dominant side appears to contribute to greater thickness of the splenius capitis on the non-dominant side of pitchers.

## 1 Introduction

Functional asymmetry, muscle imbalance, and strength asymmetry have been identified as factors associated with a higher risk of injury in both the upper and lower limbs of athletes (1–4). Asymmetry can be defined as a bilateral imbalance between a homologous group of muscles or a disruption in the agonist–antagonist ratio (2). According to motor control theory, the presence of asymmetry represents potential restraints that limit an athlete's movement strategies (4, 5). Sports that often require unilateral excursion of skilled movements to be repeated frequently during games and throughout the season, such as pitching or batting in baseball, considerably increase the chances of developing a stronger dominant side (3).

Baseball fundamentally involves throwing, batting, and catching the ball. Throwing and batting are predominantly executed by the athlete's dominant hand. Notably, pitching and batting involve explosive, fast, rotational movements that can put significant strain on the dominant side, potentially leading to overloading injuries (6).

Previous research has demonstrated shoulder and scapular asymmetries in baseball players, which appear as adaptations predominantly to their dominant arm (7–10). These adaptations manifest as increased scapular anterior tilt (9) and decreased upward rotation on the dominant side (9). In addition, studies have noted asymmetries between the strength of the rotator cuff on the dominant and non-dominant sides (8) and increased strength of the lower and middle trapezius muscle on the dominant side (7). Moreover, some recent investigations revealed that adolescent baseball pitchers exhibit greater thickness and cross-sectional area of the lower trapezius muscle in their dominant arm compared to their non-dominant arm (7, 11). Although these studies have shed light on asymmetries in the upper extremities, there is a lack of evidence on neck muscle asymmetries in baseball players. This gap in knowledge is significant due to the strong activation and changes in thickness of deep dorsal neck muscles observed during isometric shoulder contraction, particularly during maximal isometric shoulder abduction (12–14).

The increased risk of injury associated with bilateral asymmetry in muscle strength has been reported in various types of overhead athletes (3, 15), including volleyball players (16, 17). For example, Hadzic et al. found that in male volleyball players, the external to internal rotation strength ratio of the dominant shoulder was lower, regardless of the playing position or skill level. In female players, however, this ratio was reduced only in those with higher skill levels. Accordingly, they suggested that female volleyball players may have a lower risk of developing shoulder-related problems compared to their male counterparts (17). In addition, Wang and Cochrane reported that an imbalance in the external to internal rotator strength on the dominant side was significantly associated with a higher risk of injury in volleyball players (18). Although asymmetric scapular dyskinesia has been observed in volleyball players, its link to injury risk remains controversial (19, 20). Furthermore, Reeser et al. found an association between shoulder pain and asymmetric pectoralis shortness in volleyball players (16). However, there is limited evidence regarding muscle asymmetry, particularly in the neck muscles of baseball players, and whether such asymmetry is associated with an increased risk of injury for these athletes.

The significance of neck muscles lies in their pivotal role in glenohumeral biomechanics, owing to their anatomical interconnection with the shoulder. Deep neck extensor muscles are responsible for upholding neck stability and regulating the segmental movements of the cervical spine, while working with the deep neck flexors (21). Consequently, any alteration or asymmetry in this region can impact the kinetic chain during complex movements such as pitching in baseball, potentially predisposing athletes to injury.

To date, no studies have examined the impact of these repetitive unilateral arm movements on the potential asymmetry of dorsal neck muscles, specifically in terms of muscle thickness or stiffness. Therefore, the primary aim of this study was to investigate the thickness and stiffness of the dorsal neck muscles in baseball pitchers and compare them with individuals who generally engage in symmetrical activities. We hypothesized that baseball pitchers would exhibit greater thickness in their dorsal neck muscles on their dominant side, accompanied by decreased stiffness compared to their non-dominant side and compared to non-baseball players.

## 2 Material and methods

### 2.1 Participants

A cohort containing 14 collegiate baseball pitchers and 15 controls voluntarily participated in this study, providing their written informed consent. The inclusion criteria were as follows: male participants; aged 18–40 years; and without any recent history (within the past 12 months) of neck pain, trauma, injury, or surgical interventions. Individuals were excluded from participation if they reported current neck or shoulder discomfort, or engaged in sports other than baseball, or any regular unilateral sport activities such as tennis. However, individuals with a regular regimen of gym exercise were considered eligible to participate in this study. Approval for the study was obtained from the Institutional Review Board (IRB) at California State University, Los Angeles (IRB No. 1991571-1).

### 2.2 Experimental procedure

The experimental procedure was carried out during a single visit. Participants were informed about the procedure and the equipment before data collection. Anthropometric data, including height, weight, and age, were collected via demographic information sheets. In addition, participants provided information about their exercise routines, including the duration in years and frequency per week. The outcome measures included dorsal neck muscle thickness, stiffness, and strength, neck range of motion (ROM), and cervical repositioning error as an index of neck proprioception. Ultrasound imaging for muscle thickness and stiffness was conducted by the principal investigator with over 10 years of experience in ultrasound imaging. The remaining tests and measurements were performed by a physical therapist with more than 5 years of experience.

#### 2.2.1 Ultrasonography measurements

Rehabilitative ultrasound imaging (RUI) using a V7, 2020 (Samsung, Korea), equipped with a 4-cm LA2-14A linear probe, was used to measure muscle thickness and stiffness. Participants were seated with relaxed heads and necks, with their hands resting on their thighs (22). The cervical vertebra 4 (C4) was palpated and marked by a skilled physical therapist. Then, the probe was horizontally placed on the C4 spinous process and gradually slid to either the left or right side (randomized order). Once the vertebral lamina and separating fascia were clearly visible, the image was frozen to measure muscle thickness. In the aforementioned probe position, the screen displayed images of the trapezius, splenius capitis, semispinalis capitis, semispinalis cervices, and multifidus muscles. This process was repeated three times and the average thickness of each muscle on each side was used for further analysis. No normalization was done for muscle thickness as weight is the main factor influencing neck muscle size (23) and due to the similarity of weight across both groups.

Elastography settings were configured to the musculoskeletal neck preset, with a 10 Hz penetration rate and a shear modulus range of up to 600 kPa. For the stiffness measurement, the probe was adjusted vertically on C4 and slid to either the right or left side (randomized order) until a clear image was obtained. Image quality was assessed using the Relative Measurement Index (RMI). With the greenest possible RMI screen indicating optimal image quality, the image was saved. A region of interest (ROI) was manually set for each muscle, excluding the fascia and hypoechoic layers. Within each ROI, five distinct points were selected on each muscle, with the average stiffness across these points considered to be the muscle's stiffness.

#### 2.2.2 Neck proprioception

**Figure 1:**
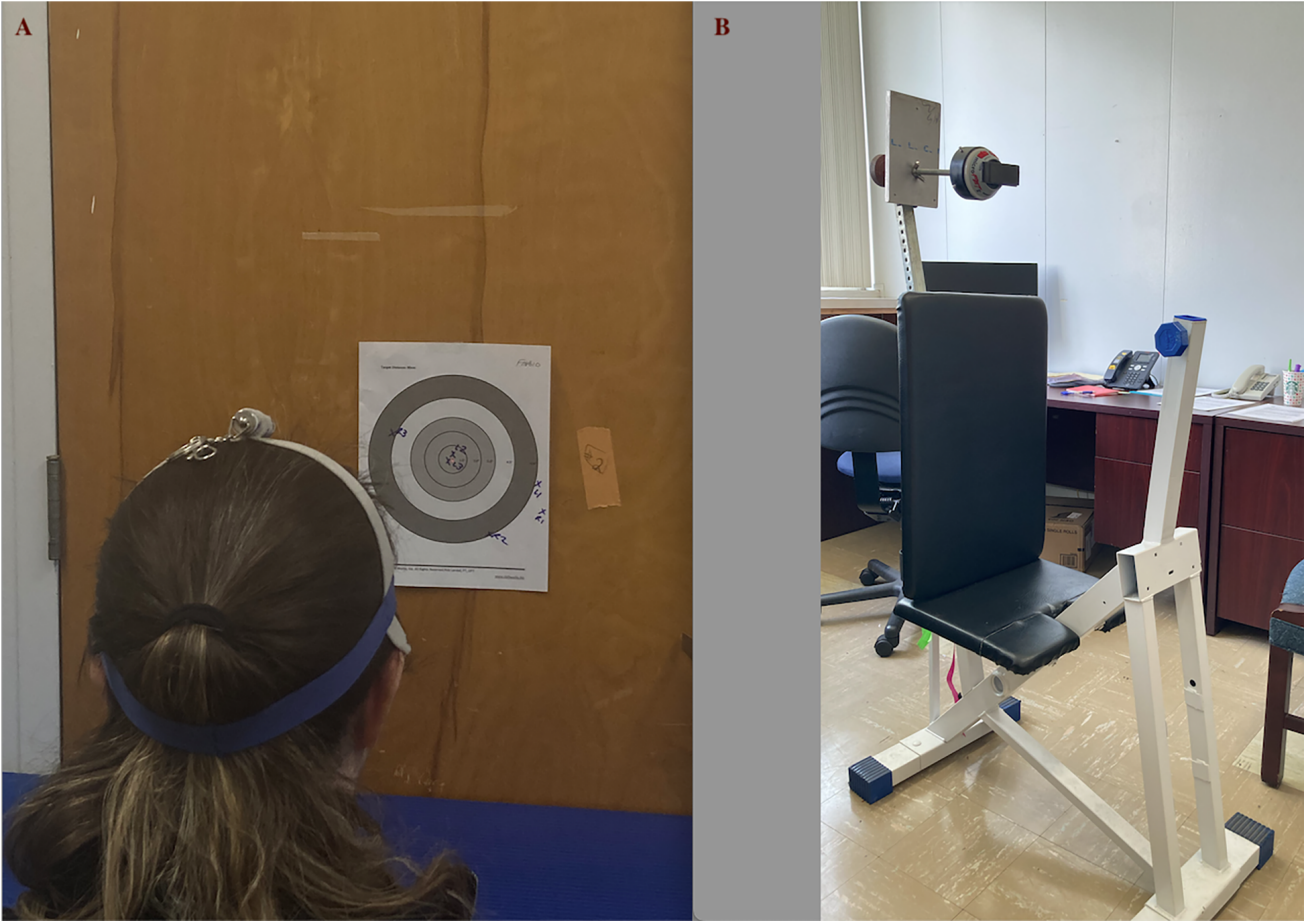
**(A)** Neck repositioning error test. **(B)** The customized chair and mounted handheld dynamometer.

Neck proprioception was assessed using the neck repositioning error test (24, 25). Participants were seated relaxed on a chair with their hands resting on their thighs. The chair was positioned at a fixed distance of 90 cm from a wall. A headband equipped with a laser pointer was affixed on the individual’s head, directing the laser light onto the wall in front of them. A target was placed on the wall at eye level. Participants were instructed to maintain a natural gaze and keep their head and neck relaxed throughout the procedure (Figure 1A).

The laser point on the wall served as the initial reference. Participants were then asked to rotate their head and neck either to the right or left (randomly selected) as far as possible before returning to starting position to familiarize themselves with the procedure. Next, they repeated the same movement sequence with their eyes closed. Upon reaching what they perceived as their original head position, the new location of the laser pointer on the wall was marked. The difference between the target point and the new marked point represented the repositioning error (24, 25).

Participants repeated this protocol on both sides (right and left), three times per side. The average error derived from the three trials on each side was utilized for further analysis. The distance error was computed as the arctangent of the average distance from the target point, normalized to the fixed distance of 90 cm to the wall. A distance error exceeding 4.5° is considered clinically important (26).

#### 2.2.3 Neck range of motion

The right and left lateral flexion and rotation movements of neck ROM were assessed using a bubble inclinometer.

For lateral flexion ROM assessment, the bubble inclinometer was positioned on the participant's head apex. Participants were seated comfortably on a chair with their hands resting on their thighs. They were instructed to move their neck to the furthest point within the available range in one of the randomly assigned directions of head/neck movements. Participants were then prompted to bend their ears toward either their right or left shoulder while ensuring their shoulders remained stationary.

To evaluate the rotation ROM, participants were positioned in a supine manner, to avoid trunk rotation, and the bubble inclinometer was placed on the forehead. They were instructed to rotate their head to the maximum extent possible on either the right or left side. Each movement direction was performed three times and the average value was computed for further analysis (27, 28).

#### 2.2.4 Neck extensor strength

Maximum voluntary contraction (MVC) of neck extension strength was assessed using a customized chair to mount a handheld dynamometer on it without it moving around (Figure 1B) (29). The handheld dynamometer (MicroFET2 Manual muscle tester; Hoggan Scientific, Salt Lake City, UT, USA) was mounted on a rod that was fitted to each participant's height. In addition, a mechanism allowed the forward and backward movement of the dynamometer to adjust its distance, ensuring the participant's neutral head position (Figure 1).

Participants were seated with relaxed heads and necks, hands resting on thighs, and a chest belt to restrict trunk and shoulder involvement. They were instructed to push the dynamometer with the back of their head for a maximum of 3 s. The procedure was repeated three times with 30-s intervals to prevent fatigue. The highest recorded force was considered as the neck extension MVC.

### 2.3 Statistical analysis

The asymmetry index was calculated as the difference between the values of each variable on the dominant and non-dominant sides. The bigger the asymmetry index, the larger the asymmetry observed between the two sides.

Normality was assessed using the Shapiro–Wilk test. To compare symmetry indices between the groups, an independent samples *t*-test was used for normally distributed data, while a Mann–Whitney test was utilized for non-normally distributed data. Within each group, the mean dominant and non-dominant side muscle stiffness and thickness were compared using a paired *t*-test. The level of significance was set at *α* = 0.05.

## 3 Results

### 3.1 Demographic information

The participants’ demographic information is outlined in Table 1. A significant age difference was observed between the two groups, with older individuals found in the control group. The mean age difference between the groups was calculated to be 4 years (*p* = 0.009). In addition, baseball players were approximately 10 cm taller than the control group (*p* < 0.001). No significant difference was observed among the two groups in terms of weight. All baseball players and 13 of the 15 controls were right-handed. All baseball players were collegiate athletes with a mean of 16.23 ± 3.03 years of experience in baseball. They were actively participating in baseball (pitching) at the time of data collection.

**Table 1:** Demographic information of participants.

	Baseball pitchers (*n* = 14)	Controls (*n* = 15)
Age (years)	21.86 ± 1.61	25.87 ± 5.10
Height (cm)	184.53 ± 8.51	174.45 ± 5.33
Weight (kg)	86.82 ± 11.47	84.55 ± 22.49
Years of experience (years)^a^	16.23 ± 3.03	7.36 ± 7.39
Exercise history (days/week)	5.21 ± 0.89	3.78 ± 1.52

^a^ “Years of experience” for the control group reflect their regular gym exercise routines, while for the baseball players, it represents their years of experience in baseball.

### 3.2 Muscle thickness

The independent samples *t*-test revealed that baseball players exhibited a significantly greater asymmetry in the splenius capitis muscle thickness compared to the control group [*p* = 0.029, effect size (ES) = 0.857]. Table 2 details the mean and standard deviation (SD) of muscle thickness in both groups. Within each group, a paired *t*-test highlighted that among baseball players, the non-dominant side splenius capitis is significantly thicker than that on the dominant side (*p* < 0.001, ES = 1.14, mean = 0.656 vs. 0.589 cm, respectively) while no significant asymmetry was observed in the control group for this muscle. No other significant asymmetry was observed in other muscles.

**Table 2:** Mean ± standard deviation of muscle thickness and stiffness in both groups.

	Side	Muscle thickness (cm)		Muscle stiffness (KPa)	
		Baseball pitchers (*n* = 14)	Controls (*n* = 15)	Baseball pitchers (*n* = 14)	Controls (*n* = 14)
Trapezius	Dominant	0.28 ± 0.08	0.24 ± 0.08	52.38 ± 31.17	60.92 ± 20.92
	Non-dominant	0.28 ± 0.07	0.33 ± 0.21	49.33 ± 24.31	78.37 ± 29.00
Splenius capitis	Dominant	**0.59** **±** **0.10**	0.70 ± 0.14	31.79 ± 14.51	41.76 ± 18.74
	Non-dominant	**0.66** **±** **0.12**	0.699 ± 0.11	38.37 ± 17.41	58.30 ± 20.14
Semispinalis capitis	Dominant	0.69 ± 0.09	0.64 ± 0.11	32.46 ± 33.91	36.19 ± 11.00
	Non-dominant	0.72 ± 0.10	0.62 ± 0.12	35.49 ± 24.89	60.24 ± 27.36
Semispinalis cervicis	Dominant	0.70 ± 0.10	0.70 ± 0.08	39.27 ± 32.46	37.50 ± 19.04
	Non-dominant	0.67 ± 0.15	0.71 ± 0.09	31.52 ± 22.55	48.23 ± 29.02
Multifidus	Dominant	0.94 ± 0.23	0.74 ± 0.11	37.03 ± 24.33	32.71 ± 11.66
	Non-dominant	0.92 ± 0.13	0.77 ± 0.15	37.47 ± 19.03	43.68 ± 25.58

The values in bold indicate where there is a significant difference.

### 3.3 Muscle stiffness

Due to low RMI of dorsal neck muscles in one participant within the control group, the measurement of muscle stiffness was considered unreliable for them. Consequently, we excluded this individual from the analysis. Independent samples *t*-tests demonstrated that the control group exhibited a greater degree of stiffness asymmetry in the splenius capitis muscle compared to baseball players (*p* = 0.006, ES = 1.179). In addition, a Mann–Whitney *U*-test showed a similar asymmetry difference between the two groups, with greater stiffness asymmetry for the semispinalis cervicis muscle in the controls (*p* = 0.043, mean rank: 10.46 vs. 16.54). However, the within-group comparisons did not demonstrate any significant side differences for either of the groups.

No other significant differences were observed for muscle stiffness asymmetry between the two groups.

### 3.4 Neck proprioception and range of motion

No significant difference in proprioception asymmetry index was found between the groups. Similarly, the neck rotation asymmetry index was found to be comparable in both groups. However, for ROM, baseball players exhibited a significantly smaller asymmetry in terms of lateral side bend with the mean difference of 6.44° (*p* = 0.045, ES = 0.8). Controls generally showed a greater lateral bend to the non-dominant side, although there was no significant difference between sides (mean difference: 1.82°). Table 3 presents the mean and SD values of neck ROM and repositioning errors.

**Table 3:** Mean ± standard deviation of neck ROM and joint repositioning error (JRE) in both groups.

		Baseball pitchers (*n* = 14)	Controls (*n* = 15)
JRE (°)	Dominant	6.00 ± 1.84	5.31 ± 2.09
	Non-dominant	5.96 ± 2.18	5.98 ± 2.77
Neck side bend ROM (°)	Dominant	53.45 ± 11.18	47.95 ± 7.91
	Non-dominant	54.36 ± 9.73	49.63 ± 7.39
Neck rotation ROM (°)	Dominant	93.68 ± 6.29	81.15 ± 10.08
	Non-dominant	95.90 ± 8.57	82.39 ± 9.67

### 3.5 Neck extensor strength

Baseball pitchers showed significantly stronger neck extensors compared to the controls. The independent samples *t*-test revealed a *p*-value of 0.017 and an ES of 0.926 for the differences in neck extensor MVC between the two groups. The mean neck extensor MVC values were 131.161 N vs. 96.653 N for baseball players and their controls, respectively.

## 4 Discussion

This study aimed to examine whether the repetitive throwing movements involved in baseball pitching induce morphological and functional changes in the dorsal neck muscles on the dominant side, which is predominantly utilized for throwing. In addition, we sought to compare the outcomes of baseball players with those of a control group.

Our results demonstrated a significantly thicker splenius capitis muscle on the non-dominant side of baseball players. The splenius capitis muscles extend the neck when contracting bilaterally and during ipsilateral side bends and when contracting unilaterally during rotation (30). In baseball, pitchers often turn their heads toward the non-dominant side to throw and to track the ball visually after releasing. Furthermore, during the cocking phase of throwing, the cervical spine extends in coordination with the trunk, maintaining neck extension to follow the ball's trajectory. Our findings can indicate that repetitive neck rotation to the non-dominant side among baseball pitchers significantly influences the thickness of the splenius capitis muscle on the non-dominant side. This suggests a potential link between splenius capitis thickness and the effects of neck rotation, rather than from the throwing motion of the dominant arm.

In addition, we observed significantly greater asymmetry in muscle stiffness for the splenius capitis and semispinalis cervicis muscles in the control group compared to the baseball pitchers. However, no significant differences between sides were found within the groups. This suggests the presence of latent trigger points without perceived pain in the controls, although the observed asymmetry was not sufficient to induce a significant asymmetry in controls. In other words, neck muscle stiffness was symmetric in both groups, although the controls showed a non-significant tendency toward asymmetry in the two abovementioned muscles.

Individuals experiencing unilateral pain typically demonstrate higher muscle stiffness on their affected side compared to the contralateral side (31, 32). Our findings show symmetrical dorsal neck muscle stiffness in both groups, suggesting that within healthy populations, neck muscle structure tends to exhibit symmetry.

We expected an asymmetry in neck ROM in baseball pitchers as they turn their heads to track the ball. However, contrary to our expectations, our study revealed symmetrical neck ROM among baseball pitchers and controls. This finding is consistent with Devaney's research, which similarly reported symmetrical ROM in baseball players (33).

This study represents the first investigation into cervical proprioception among baseball players. Previous research comparing neck repositioning errors in individuals with and without neck pain has shown reduced cervical proprioception in those with traumatic neck pain (34). The multifidus muscle contains numerous muscle spindles that play a critical role in providing accurate cervical proprioception (26). Our ultrasound investigation revealed symmetrical multifidus thickness and stiffness in both groups, corresponding to the symmetric repositioning errors observed. In addition, our control group consisted of healthy individuals without a history of neck pain, which further supports the finding of symmetric cervical proprioception in this group.

Finally, baseball pitchers exhibited significantly greater MVC for neck extensors compared to controls, which was anticipated given their extensive training regimen, including shoulder and rotator cuff strengthening exercises (35). A review by Hrysomallis in 2016 (36) has shown that athletes generally have higher neck and shoulder isometric strength when compared to non-athletes, mostly due to their rigorous training programs typically conducted three to four times a week. In addition, baseball pitchers track the ball after throwing and actively stabilize their necks against the rotating trunk, which likely contributes to increased thickness of the splenius capitis and greater strength in neck extensor muscles.

Significant differences in age and height were observed between the two groups. However, the mean ages of individuals in both groups were found to be below the threshold of 30 years, thereby mitigating the potential influence of age-related physiological variations on the study outcome (37). Furthermore, it is important to consider that neck muscle size is primarily associated with weight rather than height (23). Therefore, we believe that these differences between the two groups did not impact our results.

Our study results are interpreted with consideration of some limitations. We measured the thickness and stiffness of dorsal neck muscles at rest. However, assessing their thickness and stiffness during muscle contraction would provide valuable insights into their function and response during activities. Future studies should consider investigating these aspects. In addition, our baseball pitchers did not have a history of shoulder injury or shoulder pain, which may contribute to observing symmetric neck muscles. Nonetheless, evaluating baseball pitchers who have experienced injuries could reveal a potential link between asymmetries and risk of injury. Future studies focusing on pitchers with shoulder injuries could provide valuable insights into these aspects.

## 5 Conclusion

Baseball pitching, a repetitive unilateral activity performed by pitchers, may not be linked with unilateral development of deep dorsal neck muscles. Deep neck muscles, such as the multifidus muscle, contribute to neck stability during arm movements (13, 14). Our results indicate that despite the unilateral throwing motion in baseball pitching, both cervical multifidus muscles are involved in stabilizing the neck during arm movements. However, it appears that unilateral neck rotation toward the non-dominant side may result in greater thickness of the splenius capitis on the non-dominant side.

## Data Availability

The raw data supporting the conclusions of this article will be made available by the authors, without undue reservation.
